# Effects of *Thymbra capitata* essential oil on in vitro fermentation end-products and ruminal bacterial communities

**DOI:** 10.1038/s41598-023-31370-9

**Published:** 2023-03-13

**Authors:** María José Ranilla, Sonia Andrés, Chiara Gini, Filippo Biscarini, Cristina Saro, Alba Martín, Iván Mateos, Secundino López, F. Javier Giráldez, Latifa Abdennebi-Najar, David Pereira, Hanen Falleh, Riadh Ksouri, Paola Cremonesi, Bianca Castiglioni, Fabrizio Ceciliani

**Affiliations:** 1 Departamento de Producción Animal, Facultad de Veterinaria, Universidad de León, León, Spain; 2 Instituto de Ganadería de Montaña, CSIC-Universidad de León, Grulleros, León, Spain; 3 Department of Veterinary Medicine and Animal Sciences, Università Degli Studi di Milano, Lodi, Italy; 4 Department of Bioinformatics, Biostatistics, Genomics, IBBA-CNR, Institute of Agricultural Biology and Biotechnology, Milan, Italy; 5 Quality and Health Department, IDELE Institute, 75595 Paris Cedex 12, France; 6 Centre de Recherche Saint-Antoine (CRSA), INSERM UMR_S_938, Sorbonne University, 75020 Paris, France; 7 REQUIMTE/LAQV, Laboratory of Pharmacognosy, Department of Chemistry, Faculty of Pharmacy, University of Porto, 4050-313 Porto, Portugal; 8 Laboratory of Aromatic and Medicinal Plants, Biotechnology Center of Borj-Cédria, BP 901, 2050 Hammam-Lif, Tunisia

**Keywords:** Metagenomics, Microbial ecology

## Abstract

An in vitro trial was carried out to investigate the effects of natural *Thymbra capitata* essential oil (NEO) and its main compounds [including carvacrol, *p*-cymene, *γ*-terpinene given alone or in a synthetic combination (SEO)] on ruminal fermentation and the bacterial community using batch cultures inoculated with ruminal digesta and incubating two different basal diets [high-forage (F) and high-concentrate (C) diet]. After 24 h of incubation, primary fermentation end-products [gas, methane, volatile fatty acids (VFAs) and ammonia] and rumen microbial diversity were determined. NEO reduced the total VFA concentration (*P* < 0.05) only in the C diet. In contrast, SEO and carvacrol decreased the total VFA concentration (*P* < 0.05) only in the F diet. Methane production was not affected (*P* > 0.05) by any of the experimental treatments or diets evaluated. Microbial diversity analysis showed only a moderate effect of carvacrol and SEO on 13 genera, including, mainly, *Atopobium* and *Blautia* (involved in subacute ruminal acidosis) or *Candidatus Saccharimonas* (related to laminitis). In conclusion, *T. capitata* EO has a limited potential to attain nutritional or environmental benefits, but further research should be carried out to clarify its effects on animal health and microbial food safety.

## Introduction

Over the last few decades, many efforts have been made to investigate alternative substances to antibiotics as growth promoters in animal farming^1^. Plant secondary metabolites present in essential oils (EOs), including mainly terpenes and phenylpropanoids, have shown a marked antimicrobial effect through mainly (but not only) increasing the fluidity and permeability of the cytoplasmic membrane, alteration of ion gradients, depletion of intracellular ATP concentration or inactivation of bacterial enzymes^2^. These effects on microbial cells may cause bacterial lysis, especially in the case of Gram-positive bacteria, in which cell membranes can interact directly with hydrophobic compounds of EOs. On the contrary, the outer membrane of Gram-negative bacteria (hydrophilic) acts as a natural barrier against these lipophilic compounds, but compounds with smaller molecular size (e.g., carvacrol or thymol) can still pass through porin proteins to access the cytoplasmic membrane^2,3^. The sensitivity of microbes to EOs varies depending on all these factors, which justifies the interest in these compounds in ruminant nutrition. In fact, the effects on the microbial population may have an impact on rumen fermentation processes, including less methane production, decreased degradation of feed protein or increased feed efficiency, via selection for or against specific groups of microorganisms in the rumen^4^.

The composition of EOs varies depending on many factors, including the phenological stage or part of the plant, geographical area and extraction procedures^3^. Therefore, a proper characterisation of the effects promoted by the different active compounds included in EOs is required to take advantage of the full potential of the EOs in terms of health benefit or nutritional or environmental impacts when included in the diet of ruminants. There is evidence of the broad spectrum of activity of thymol and carvacrol (phenolic compounds present in EOs obtained from *Origanum, Thymus* or other plant species of the *Lamiaceae* family) against a great variety of Gram-positive and Gram-negative bacteria. Most of these studies have shown that high concentrations of both thymol and carvacrol (> 225 mg/L), despite partially depressing rumen methanogenesis, may non-selectively inhibit rumen fermentation, which is not beneficial nutritionally for the host animal. A low-molecular-weight of these compounds may allow them to slowly cross the cell wall by diffusion through the lipopolysaccharides layer or membrane proteins and interact with the lipid bilayer of cells, thus reducing their selectivity against specific populations^2^.

On the other hand, other compounds with low antimicrobial activity, such as *p*-cymene, have reduced methanogenic activity at low concentrations (20 mg/L) without adversely affecting ruminal fermentation, probably due to its more specific effects (compared with thymol or carvacrol) on rumen methanogens^3^. Therefore, more systematic studies attending to the effects of the pure compounds (or combinations) are needed for the most promising EOs in animal nutrition. Consequently, this study was carried out to investigate the effects of a well-characterised EO (known phytochemical profile and pure bioactive compounds) obtained from *Thymbra capitata* on the ruminal bacterial community and in vitro fermentation end-products. The main objectives of this study were (1) testing/evaluating the effects of EOs (and their components) on rumen fermentation end-products using batch cultures inoculated with ruminal digesta and incubating either a high forage (F) or a high concentrate (C) diet, and (2) testing the effects of each pure bioactive compound on ruminal bacterial community composition and diversity.

## Methods

### Plant material, *Thymbra capitata* essential oil, synthetic essential oil, and pure bioactive compounds

About 3 kg of *Thymbra capitata* (L.) Cav. (Mediterranean or cone-head thyme, for which the former scientific name *Thymus capitatus* (L.) Hoffmanns. & Link is a synonym) aerial parts were collected at the vegetative stage from a naturally diversified mountain in the Northeast of Tunisia (27 m above sea level, 37° 02′ N, 10° 59′ E). All samples were shade-dried for 15 days at room temperature. The dried material (200 g) was ground, and the natural essential oil (NEO) was extracted as described previously by BenJemaa et al.^5^ The collection of *Thymbra capitata* shoots (e.g., leaves and stems at the vegetative stage) and the extraction of its essential oil complied with the national guidelines of Tunisia. A gas chromatograph (HP 5890-SERIE II, Agilent, Waldbronn, Germany) coupled to a mass spectrometer (HP-MSD 5972 A, Agilent, Waldbronn, Germany) equipped with an HP INNOWAX polar column (30 m × 0.25 mm; film thickness, 0.25 µm) was used to analyse the chemical composition of *Thymbra capitata* NEO. The oven temperature was programmed to rise from 50 to 240 °C at a rate of 5 °C/min. The transfer line temperature was 250 °C. The carrier gas was helium at a flow rate of 1.2 mL/min; the split ratio was 60:1. Scan time and mass range were 1 s and 40–300 m/z, respectively. The NEO volatile compounds were identified by comparing their retention index (RI) values related to (C9–C18) *n*-alkanes with those of authentic compounds (Analytical reagents, LabScan, Ltd, Dublin, Ireland) available in the literature and by matching their mass spectrum fragmentation patterns with corresponding data stored in the mass spectrum library of the GC–MS data system (NIST) and other published mass spectra. Relative percentage amounts of the identified compounds were obtained from the electronic integration of the FID peak area. Percentages of compounds (carvacrol 70.62%; *p*-cymene 7.06%; γ-terpinene 7.58%) were calculated using the HP-ChemStation software based on their respective areas. Further details about the *Thymbra capitata*** (**NEO) used in the present study can be found in BenJemaa et al.^5^.

The three significant compounds detected in *Thymbra capitata* NEO were then purchased from Sigma Aldrich and used to formulate a synthetic essential oil (SEO) with the same composition determined in the NEO (carvacrol 70.62%; *p*-cymene 7.06%; γ-terpinene 7.58%).

### Ruminal incubation in batch cultures

The effects of *Thymbra capitata* NEO and its main compounds either alone (carvacrol, *p*-cymene and γ-terpinene) or in combination (SEO: a synthetic combination of carvacrol, *p*-cymene and γ-terpinene) on the in vitro ruminal fermentation of either a high-forage (F; 70% forage and 30% concentrate) or a high-concentrate diet (H; 30% forage and 70% concentrate) were investigated using batch cultures of mixed ruminal microorganisms. These diets (F and C) were obtained mixing dried and milled alfalfa (10.6% ash, 32.7% neutral detergent fibre –NDF– and 4.86% acid detergent lignin –ADL–) with dried and milled concentrate feed (7.98% ashes, 12.1% NDF and 0.258% ADL) in the proportions mentioned above. The CP content and metabolizable energy (ME) of these diets were also analysed (F diet: 195 g CP/kg DM and 2.28 Mcal/kg DM, and C diet: 184 CP/kg DM and 2.73 Mcal/kg DM).

The rumen fluids for incubations were collected at the slaughterhouse from four different Holstein cows. Batch cultures were prepared by mixing 10 mL of the filtrated bovine rumen fluid with 40 mL of the buffer and incubated with tested doses of EOs or pure compounds in 120-mL vials containing 0.5 g DM of the corresponding diet (F or C). Five different experimental treatments were tested against a control (no additive), namely: (1) natural essential oil of *Thymbra capitata* (NEO) added at a concentration to provide 75 mg carvacrol/L (a concentration without inhibiting effects on ruminal fermentation in previous studies; Martín et al.^29^). Three pure compounds [(2) carvacrol, (3) *p*-cymene and (4) γ-terpinene] were added to the vials to mimic the concentration in NEO treatment (carvacrol 70.62%; *p*-cymene 7.06%; γ-terpinene 7.58%) when added at a rate of 75 mg carvacrol/mL. Finally, (5) a synthetic essential oil (SEO) [formulated using the three pure compounds according to their proportions in NEO (carvacrol 70.62%; *p*-cymene 7.06%; γ-terpinene 7.58%)] was added to the vials at a concentration of 75 mg carvacrol/mL. The EOs and pure compounds were dissolved in ethanol (30 µL of the corresponding solution *per* bottle), and control bottles received 30 µL of ethanol without any other compound. Bottles were sealed with rubber stoppers and aluminium caps and incubated for 24 h at 39 °C. Therefore, four inocula obtained from four different cows were incubated with two diets (F or C) and five experimental groups *plus* a control. Two replicates *per* group were assayed, and the total number of vials assayed was 96 (*plus* 8 more vials with no diet used as blanks).

Total gas production was measured using a calibrated syringe and a pressure transducer (Delta Ohm DTP704-2BGI, Herter Instruments SL, Barcelona, Spain). Before analysis for methane, a gas sample (10 mL) was taken in a Vacutainer (Terumo Europe NNN, Leuven, Belgium). Bottles were swirled in ice to stop the fermentation and opened. Subsequently, 1 mL of the content was added to 1 mL of 0.5 N HCl for NH_3_-N analysis, and 0.8 mL was added to 0.5 mL of deproteinising solution (metaphosphoric acid [20 g/L] and crotonic acid [0.06 g/L]) for volatile fatty acid (VFA) determination. Concentrations of ammonia-N and VFA (e.g., acetate, propionate, butyrate, valerate, caproate, isoacids) were determined as described by Carro et al.^6^, and CH_4_ was analysed by gas chromatography as described by Martínez et al.^7^.

### Sampling and sequencing the rumen microbiota

Contents (both liquid and solid phase) of the vials from diet F (but not from diet C vials, as explained below) were collected, freeze-dried, stored at − 80 °C and used for microbial DNA extraction employing the QIAamp Fast DNA Stool Mini Kit (Qiagen) according to the manufacturer’s instructions. The DNA quality (A260/280 ratio = 1.92 ± 0.15) and quantity (56.8 ng/µL ± 18.2 ng/µL) were assessed after extraction using a Nanodrop ND-1000 spectrophotometer (NanoDrop Technologies, Wilmington, DE, USA), and the isolated DNA was stored at − 20 °C until use.

Bacterial DNA was amplified by targeting the V3–V4 hypervariable regions of the 16S rRNA gene^8^. The nucleotide sequence (forward and reverse) of the primers used for the V3-V4 amplification were 5′-CCTACGGGNGGCWGCAG-3′ as forward and 5′-GACTACHVGGGTATCTAATCC-3′ as reverse. The PCR amplification of each sample was performed in a 25-μL volume, using 12.5 μL of KAPA HIFI Master Mix 2 × (Kapa Biosystems, Inc., MA, USA). Then, 0.2 μL of each primer (100 μM) was added to 2 μL of genomic DNA (5 ng/μL). Non-DNA templates (blank controls) were included. Amplification and library quantification were carried out as described previously^9^.

### Bioinformatic processing and statistical analysis

After demultiplexing, paired-end reads from 16S rRNA-gene sequencing were quality-checked using the software package MultiQC^10^. Subsequently, barcodes were removed before joining forward and reverse reads into single reads with the C++ package SeqPrep. Joined reads were filtered for quality based on the following parameters: (1) a maximum of three consecutive low-quality base calls (Phred < 19); (2) no "N"-labelled (unidentified) bases allowed; (3) at least 75% of consecutive bases in a read with Phred > 19. Sequences not matching filtering requirements were discarded. The remaining reads were combined in a single FASTA file and aligned against the SILVA database v. 132 for closed-reference OTU identification and quantification, with 97% cluster identity^11–13^. Taxonomic identification at the phylum to genus levels was obtained via a predefined map of reference sequences to each taxon. Quantified and classified OTUs were assembled into an OTU table from which OTUs with a total count lower than 10 in less than two samples were discarded. Before downstream analysis, OTU counts were normalised for uneven sequencing depth by cumulative sum scaling (CSS)^14^. The command lines used for the analysis were adapted from Biscarini et al.^15^.

### *Firmicutes:Bacteroidetes* ratio (F:B), alpha- and beta-diversity indices

The F:B ratio was calculated to check the influence of the dietary treatments on ruminal microbiota^16^. Rumen digesta microbial diversity was assessed within (alpha-diversity) and across (beta-diversity) samples. Besides the number of observed OTUs directly counted from the OTU table, within-sample microbial richness and diversity were estimated using the following indices: Chao1 and ACE (abundance-based coverage estimator) for richness, Shannon, Simpson and Fisher’s alpha for diversity^17–22^, as well as Simpson E and Pielou’s J (Shannon’s evenness) for evenness^23^. Across-sample rumen digesta microbiota diversity was quantified by calculating unweighted UniFrac distances^24^. Among groups (*T. capitata* NEO, *T. capitata* SEO, carvacrol, *p*-cymene, γ-terpinene, control) and pairwise distances were evaluated non-parametrically using the permutational analysis of the variance approach [999 permutations]^25^. The calculation of the mentioned alpha- and beta-diversity indices can be found in Biscarini et al.^15^. All data (fermentation end-products, OTU abundances, F:B ratio, diversity indices) were subjected to one-way ANOVA using the following linear model, presented as Eq. (1)

1 $$Y_{{{\text{ij}}}} =\upmu + T_{{\text{i}}} + C_{{\text{j}}} + e_{{{\text{ij}}}} ,$$

where *y*_ij_ is the individual value for each variable, *T*_i_ is the fixed effect of the *i* treatment (any of the six groups (control and five treatments) tested, *i* = 1…6), *C*_j_ is the block effect of the inoculum (cow; *j* = 1…4), and *e*_ij_ is the residual error. For fermentation end-products, the Dunnett test was used to compare each experimental treatment against the negative control (with no EO or compound added). In the case of the F:B ratio, this variable was also analysed non-parametrically via bootstrapping^26^ by taking 10,000 random samples of the original data with replacement. Bootstrapped data were used to recalculate F:B ratios, averages and variability by treatment.

### Software

Reads from 16S rRNA-gene sequencing were processed with QIIME v. 1.9^8^, using a pipeline adapted from Biscarini et al.^15^. The ACE alpha-diversity index was estimated using a custom Python script (https://github.com/filippob/Rare-OTUs-ACE), and R was employed to generate plots and figures using the ggplot2 R package^27^. Additional data handling was performed in the R environment for statistical computing^28^.

## Results

### In vitro ruminal fermentation

Neither total gas production nor methane production were significantly modified by the F diet (*P* > 0.05). However, the molar proportion of propionate was decreased when NEO was incubated with the F diet (*P* < 0.05), and the same was observed for carvacrol (Table 1). Moreover, NEO, SEO and carvacrol also gave rise to increased caproate proportion (*P* < 0.001) and an acetate-to-propionate ratio (Ac: Pr) for the F diet (*P* = 0.021). On the contrary, NH_3_-N was significantly increased with NEO and SEO but not with carvacrol.

**Table 1:** Effects of *Thymbra capitata* essential oil (natural –NEO– and synthetic –SEO–) and their main pure bioactive compounds (included at the same concentration as when EO is added at a rate of 75 mg carvacrol/L) on ruminal fermentation end-products after 24 h in vitro incubation of a high forage diet in batch cultures with mixed rumen microorganisms.

	Control	NEO	SEO	carvacrol	*p*-cymene	γ-terpinene	SEM	*P* value
Gas (mL)	146	140	139	139	140	141	1.92	0.222
Methane (µmol)	579	583	516	507	488	528	29.42	0.181
Total VFA (µmol)	2662	2579	2495*	2459*	2621	2629	40.00	0.016
Molar proportions (µmol/100 µmol)								
Acetate	66.0	66.4	65.7	66.1	66.4	66.1	0.291	0.494
Propionate	15.0	13.6*	14.2	13.8*	14.2	14.4	0.239	0.015
Butyrate	12.3	12.9	13.05*	12.9	12.8	12.6	0.170	0.081
Valerate	2.14	2.23	2.24*	2.32*	2.21	2.23	0.024	0.004
Caproate	2.22	2.78*	2.73*	2.76*	2.30	2.36	0.077	< 0.001
Isoacids	2.35	2.09*	2.15	2.21	2.17	2.29	0.052	0.032
Ac:Pr (mol/mol)	4.78	5.37*	5.22*	5.30*	5.15	5.13	0.106	0.021
Methane/VFA (mol/mol)	0.217	0.226	0.205	0.207	0.185	0.202	0.013	0.381
NH_3_-N (mg/L)	235	259*	265*	225	225	217	5.584	< 0.001

*SEM* standard error of the mean.

*Mean values marked with an asterisk are significantly (*P* < 0.05) different from the Control.

When the C diet was incubated, only subtle effects were observed (Table 2). A significant increase in gas production was observed for both carvacrol and *p*-cymene (*P* < 0.05), with no differences in methane production for any of the treatments assayed. However, the total VFA production was reduced (*P* < 0.05) for NEO when compared to the control, mainly because of a lower proportion of acetate (Table 2, *P* = 0.056). The caproate proportion was sharply increased when NEO and SEO were incubated with the C diet (*P* = 0.014). No significant effects were observed for the remaining end-products when the C diet was incubated (*P* > 0.05).

**Table 2:** Effects of *Thymbra capitata* essential oil (natural –NEO– and synthetic –SEO–) and their main pure bioactive compounds (included at the same concentration as when EO is added at a rate of 75 mg carvacrol/L) on ruminal fermentation end-products after 24 h in vitro incubation of a high concentrate diet in batch cultures with mixed rumen microorganisms.

	Control	NEO	SEO	carvacrol	*p*-cymene	γ-terpinene	SEM	*P* value
Gas (mL)	146	144	144	150*	150*	149	0.82	< 0.001
Methane (µmol)	586	564	538	554	601	569	45.82	0.937
Total VFA (µmol)	2675	2529*	2628	2679	2692	2575	33.50	0.020
Molar proportions (µmol/100 µmol)								
Acetate	59.5	57.8*	59.4	59.5	59.6	58.7	0.421	0.056
Propionate	13.7	12.2	12.0	12.4	13.0	12.9	0.575	0.374
Butyrate	18.5	19.6	18.7	18.3	18.6	19.0	0.370	0.281
Valerate	2.29	2.49	2.43	2.46	2.34	2.40	0.054	0.152
Caproate	3.98	6.02*	5.61*	5.40	4.42	4.98	0.373	0.014
Isoacids	2.03	1.88	1.90	1.87	1.99	1.97	0.048	0.153
Ac:Pr (mol/mol)	5.14	5.75	5.87	5.69	5.48	5.47	0.28	0.518
Methane/VFA (mol/mol)	0.223	0.228	0.204	0.207	0.225	0.225	0.017	0.857
NH_3_-N (mg/L)	242	255	244	244	249	266	9.82	0.533

*SEM* standard error of the mean.

*Mean values marked with an asterisk are significantly (*P* < 0.05) different from Control.

Considering the results obtained from the in vitro ruminal fermentation, only samples from the F diet (more significant differences for most of the parameters studied) were collected and subjected to sequencing for further analysis.

### Microbiome determination

#### Sequencing of the V3–V4 region

Sequencing the V3–V4 regions of the bacterial 16S rRNA genes of the 48 rumen samples produced 3,530,792 assembled reads (joined R1–R2 paired-end reads). After quality filtering, 406,972 sequences were removed, leaving 3,123,820 sequences for subsequent analyses (88.5% average retention rate, maximum 98.5%, minimum 82.7%). On average, there were 87,946 (± 14,058) sequences *per* sample in the control group, 91,782 ± 12,332 in the natural essential oil group (NEO), 47,837 (± 23,640) in the synthetic essential oil group (SEO), 56,327 ± 33,996 in the carvacrol group, 52,648 ± 34,110 in the *p*-cymene group and 53,935 ± 16,207 in the γ-terpinene group. The initial number of OTUs identified was 8,906; after filtering out OTUs with less than 10 counts in at least two samples, 2,936 distinct OTUs were left.

#### Description of the rumen core microbiota at phylum and genus level

**Figure 1:**
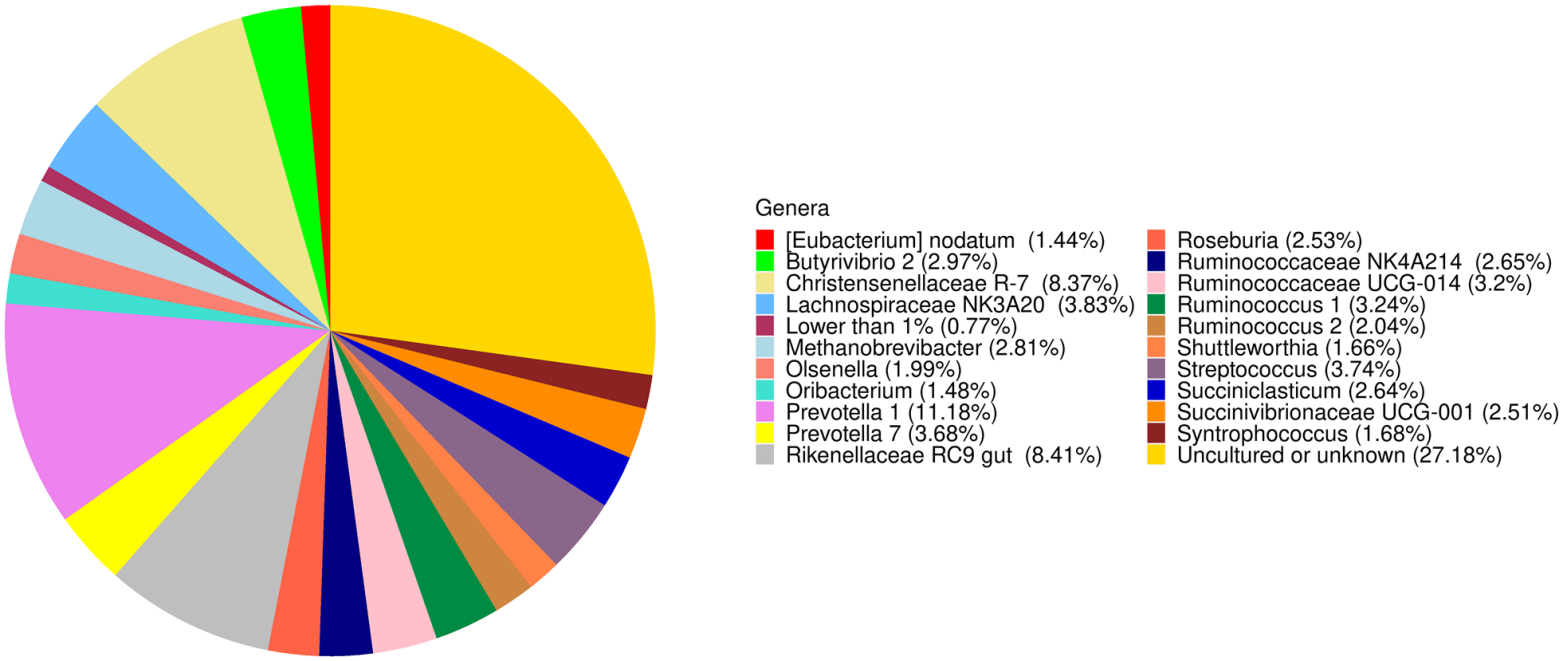
Description of the in vitro core rumen microbiota. Piechart of the relative abundance of OTUs at genera level in the rumen core microbiota (OTUs shared by 100% of the samples). Genera with relative abundance < 1% have been grouped into "Lower than 1%".

Seven main phyla were detected in the rumen microbiota, with a relative abundance larger than 1% (see Supplementary Fig. S1 online), including Firmicutes (57.46%), Bacteroidetes (25.14%) and Proteobacteria (8.3%). The other 12 phyla relative abundances below 1% and were grouped into "Other" (see Supplementary Fig. S1 online). At the genus level, 21 genera with a relative abundance greater than 1% (Fig. 1) were detected in the rumen microbiota, including *Prevotella* 1 (3.68%), *Christensenellaceae* R-7 group (8.37%), *Rikenellaceae RC9 gut group* (8.41%), *Prevotella 7* (3.68%), *Streptococcus* (3.74%), *Lachnospiraceae NK3A20* (3.83%) and *Methanobrevibacter* (2.81%). The "uncultured or unknown" group (27.18% of core rumen microbiota) was artificially composed for statistical analysis, comprehending all the genera that the database (SILVA v.132) retrieved as "uncultured" or "uncultured bacterium" or "Other" or "uncultured organism" and similar. In conclusion, the core rumen microbiota comprised 307 recognised OTUs shared by 100% of the samples *plus* the ones defined as "uncultured or unknown" as described above (see Supplementary Table 1 online).

#### Alpha- and beta-diversity indices

**Figure 2:**
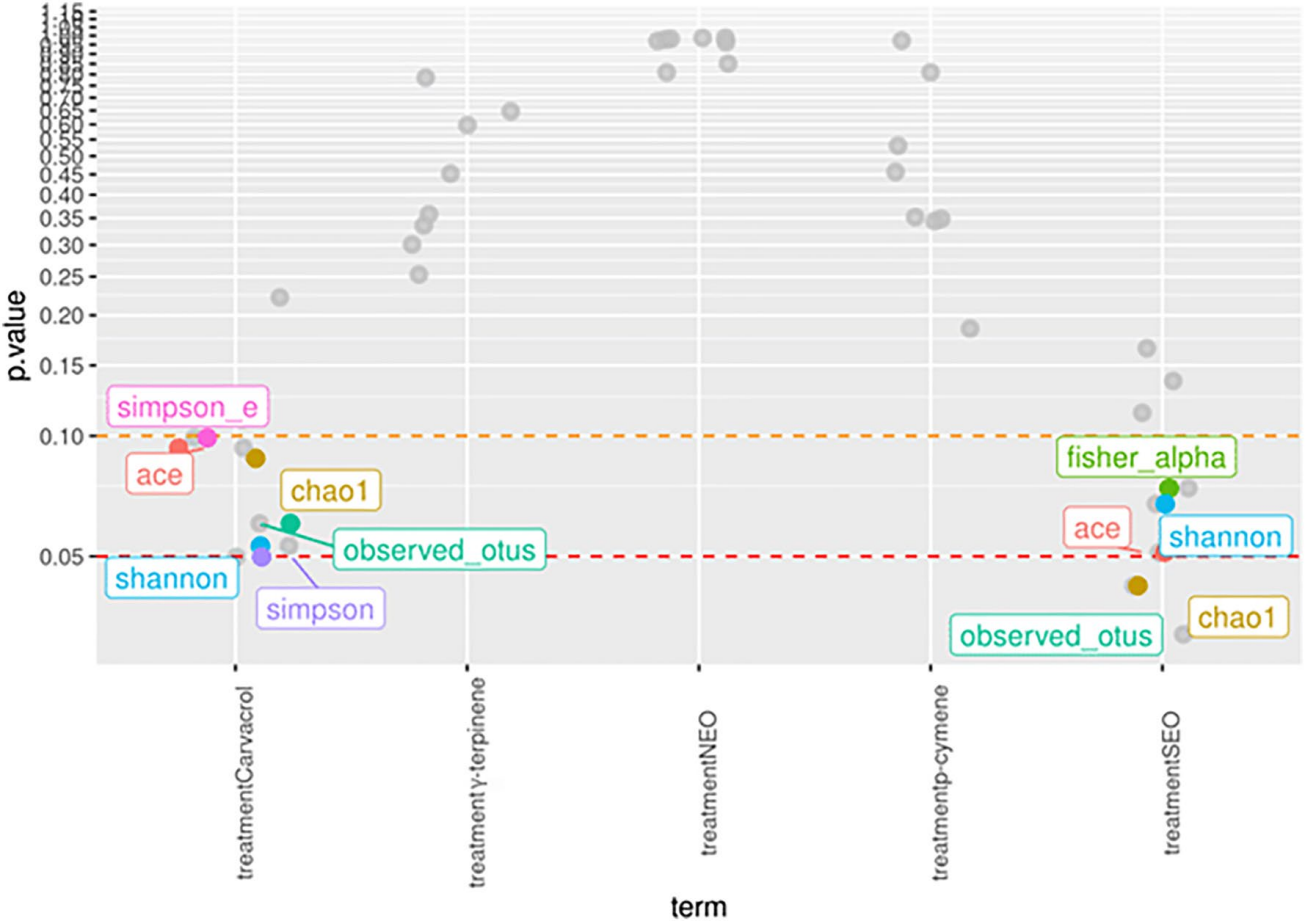
Significance of the differences between treatments for the alpha diversity indices calculated in this study. *P* values were obtained from a linear regression model with the Control group as the benchmark. The dashed lines identify the suggestive-significance area.

**Figure 3:**
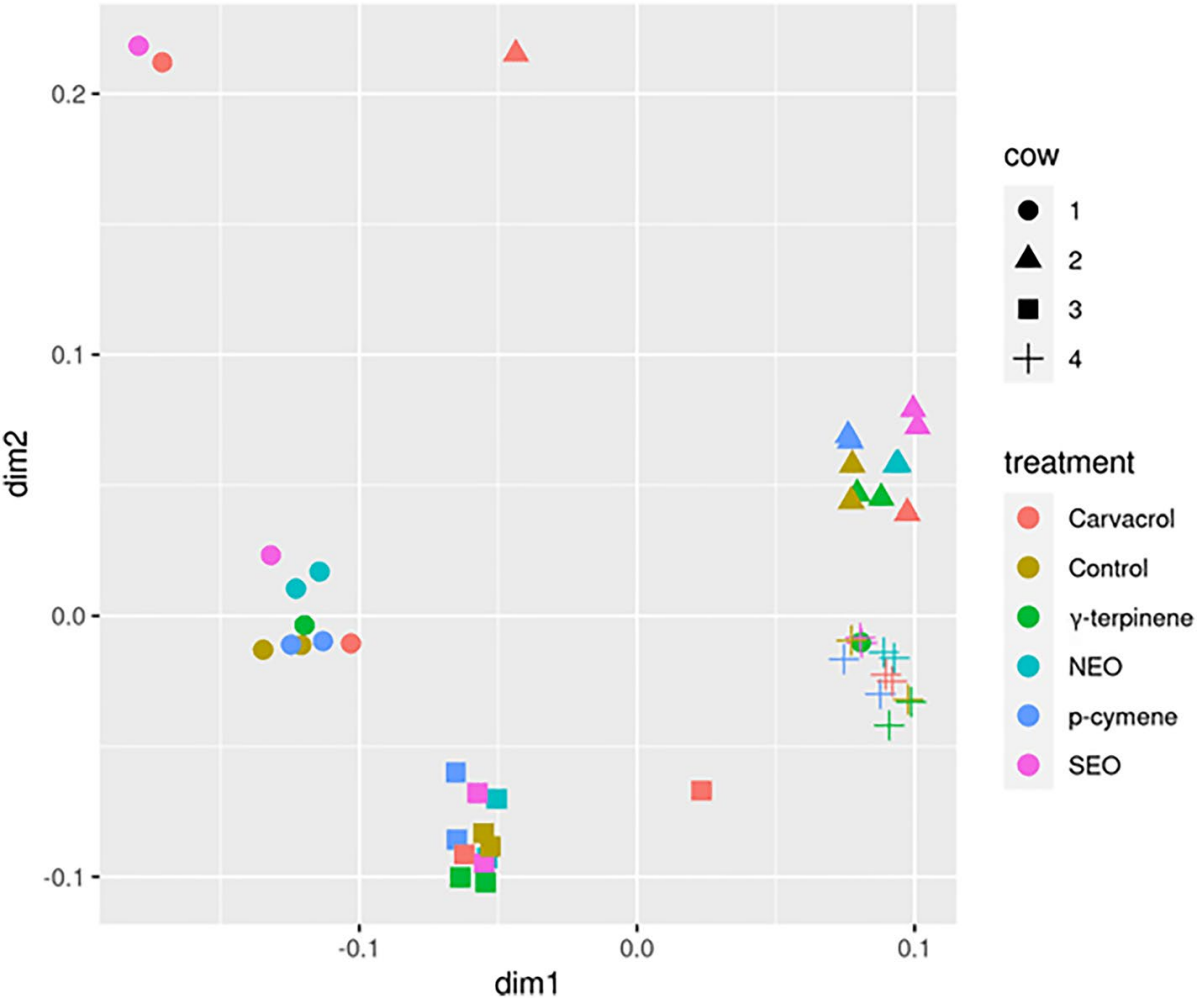
Multidimensional scaling (MDS) plot of unweighted Unifrac distances based on the OTU table from the rumen microbiota. The shape of data points represents different cows from where the rumen liquor was sampled. Colours represent the different dietary supplementations.

The estimated alpha-diversity indices for describing the richness, diversity and evenness of the rumen microbiota in the six experimental groups are shown in Supplementary Table 2 (online). Figure 2 presents the significance of the differences between treatments for the alpha-diversity indices. The carvacrol and SEO treatments differed significantly (*P* < 0.05) from the control in some of the diversity indices (Simpson, Shannon, ACE, Chao1 and observed OTUs). On the contrary, NEO, γ-terpinene and *p*-cymene treatments showed no significant (*P* > 0.20) differences compared with the control. The associations among samples were assessed based on the unweighted Unifrac distances from the beta-diversity analysis. Figure 3 shows the distribution of samples along the first two dimensions from the multidimensional scaling (MDS) of unweighted Unifrac distances: clear clusters were observed for the four cow ruminal inocula used in the experiment. In contrast, no clustering by treatment was detected.

#### Differentially abundant taxa

**Figure 4:**
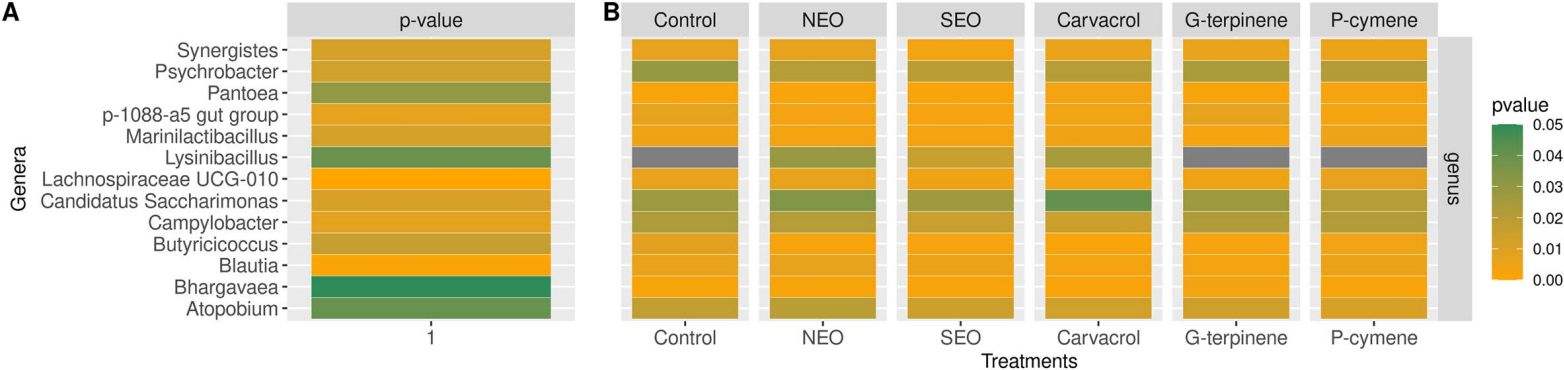
Significantly abundant taxa at genera level in the rumen microbiota: (**A**) heatmap of the *p* values of taxa relative abundance in the rumen microbiota (all 48 samples together). (**B**) relative abundance of the differentially abundant taxa (39 in total).

Comparing the control group against the five treatments highlighted significant differences in the abundance of several bacterial taxa (listed in Table 3 and Supplementary Table 3 (online)), including 5 phyla, 5 classes, 7 orders, 9 families and 13 genera. As shown in Table 3, there were significant (*P* < 0.01) differences in three genera: *Bhargavaea* (significantly increased with γ-terpinene), *Atopobium* (significantly reduced with *p*-cymene) and *Lysinibacillus* (significantly reduced with carvacrol, NEO and SEO compared to the control). Another nine genera were significantly modified (*P* < 0.05): *Butyricicoccus* (significantly reduced with all treatments except *p*-cymene), *Lachnospiraceae UCG-010* (significantly decreased with carvacrol), *Pantoea* and *Psychrobacter* (two gamma-proteobacteria significantly increased with carvacrol)*, Synergistes* (significantly reduced with SEO) and *Marinilactibacillus* (significantly reduced with γ-terpinene), *Candidatus Saccharimonas* (significantly increased with carvacrol), *Campylobacer* (significantly reduced with carvacrol and SEO) *p-1088-a5 gut group* (significantly decreased with NEO and SEO) and *Blautia* (significantly reduced with carvacrol and γ-terpinene) (Table 3, Fig. 4).

**Table 3:** Differentially abundant taxa by treatment.

Genera	Control	carvacrol	*P* value	γ-terpinene	*P* value	NEO	*P* value	*p*-cymene	*P* value	SEO	*P* value	Overall *P* value
	Relative abundance (%)	Relative abundance (%)		Relative abundance (%)		Relative abundance (%)		Relative abundance (%)		Relative abundance (%)		
*Bhargavaea*	0.000	0.000	1.000	0.022	**0.001**	0.000	1.000	0.000	1.000	0.000	1.000	**0.006**
*Atopobium*	0.205	0.215	0.066	0.170	0.111	0.234	0.370	0.137	**0.008**	0.200	0.195	**0.009**
*Lysinibacillus*	1.149	0.359	**0.005**	0.811	0.115	0.377	**0.009**	1.081	0.472	0.261	**0.002**	**0.009**
*Pantoea*	0.000	0.023	**0.003**	0.000	1.000	0.000	1.000	0.012	0.260	0.000	1.000	**0.013**
*Butyricicoccus*	0.086	0.000	**0.003**	0.010	**0.007**	0.003	**0.004**	0.047	0.059	0.016	**0.011**	**0.025**
*Psychrobacter*	0.363	0.417	**0.007**	0.334	0.080	0.246	**0.006**	0.300	**0.012**	0.375	**0.002**	**0.028**
*Marinilactibacillus*	0.034	0.056	0.666	0.009	**0.012**	0.019	0.141	0.047	0.723	0.016	0.056	**0.029**
*Synergistes*	0.070	0.582	0.321	0.058	0.252	0.675	0.850	0.049	0.099	0.025	**0.002**	**0.030**
*Candidatus Saccharimonas*	0.335	0.754	**0.035**	0.368	0.947	0.420	0.292	0.279	0.227	0.529	0.883	**0.031**
*Campylobacter*	0.278	0.167	**0.008**	0.295	0.759	0.247	0.484	0.305	0.601	0.215	**0.024**	**0.036**
*p-1088-a5 gut group*	0.065	0.028	0.073	0.073	0.914	0.021	**0.047**	0.025	0.454	0.010	**0.014**	**0.036**
*Blautia*	0.054	0.018	**0.037**	0.017	**0.025**	0.061	0.752	0.053	0.700	0.051	0.524	**0.047**
*Lachnospiraceae UCG-010*	0.062	0.023	**0.033**	0.038	0.150	0.067	0.814	0.083	0.736	0.040	0.114	**0.050**

The table presents the counts and *P* values for every genus with a significant differential abundance after different compounds’ co-incubation. *P* values are presented for each treatment against Control, and overall *P* value represents the significance of the influence of the treatments against the null hypothesis that no counts would have not to be influenced by treatment.

Significant values are in bold.

#### F:B ratio

**Figure 5:**
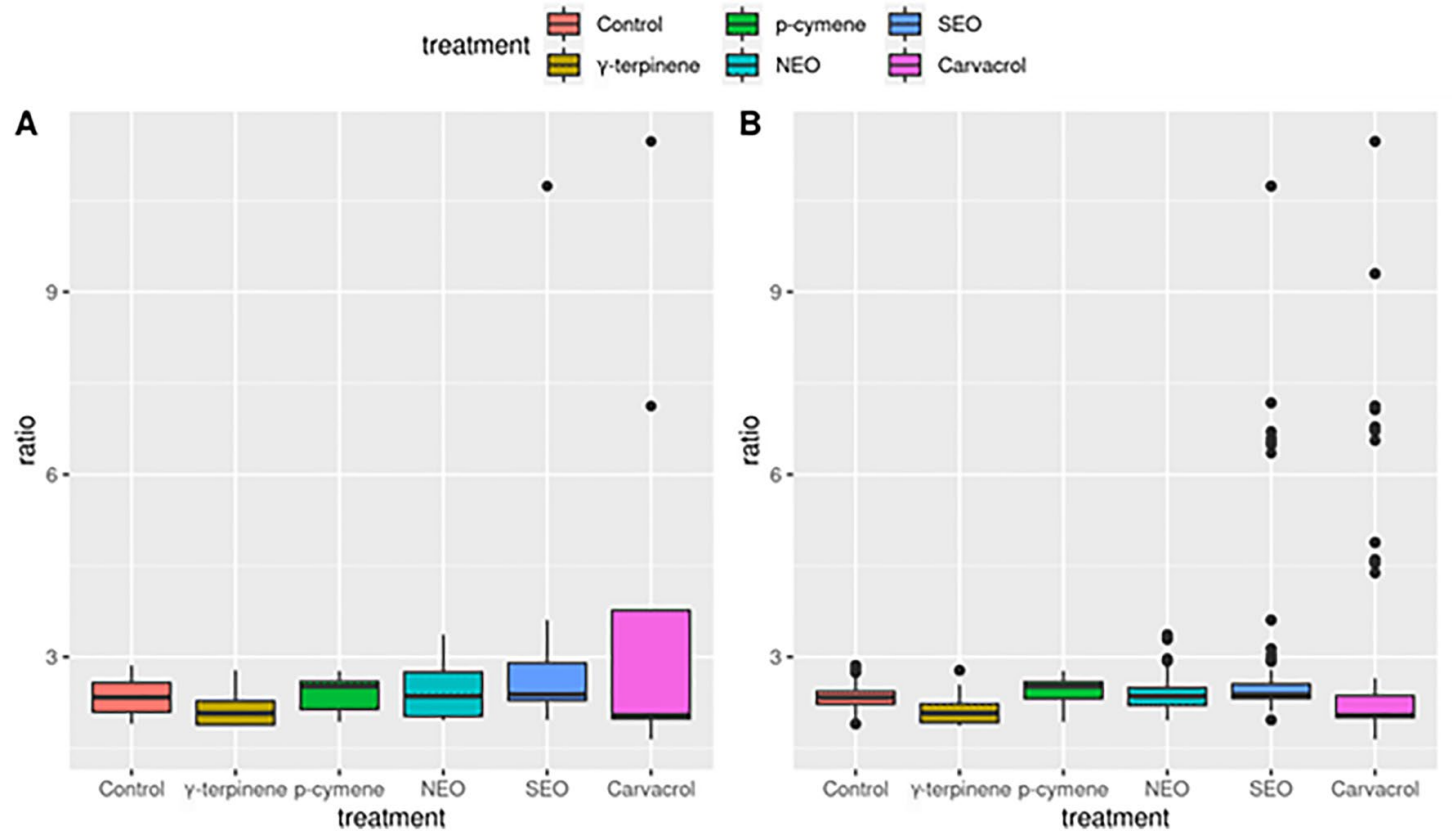
F:B ratio (*Firmicutes* to *Bacteroidetes*) in the rumen microbiota with and without dietary supplementation with essential oils. (**A**) average F:B ratio from the original data; (**B**) F:B ratio from 10,000 bootstrapping replicates.

The Firmicutes-to-Bacteroidetes (F:B) ratio was calculated to assess the influence of the dietary treatments on ruminal microbiota. Analysis of variance and bootstrapping analysis showed that the different treatments tested did not significantly change (*P* > 0.05) the F:B ratio (Fig. 5 and Supplementary Table 4 online).

## Discussion

The concentration of NEO used in this study (75 mg of carvacrol/L) was selected considering the results observed in a previous dose–response trial in which NEO showed some modulating non-inhibitory effects on ruminal fermentation^29^. Therefore, all the treatments assayed were defined to mimic the concentration of the compounds present in NEO when added to the vials at this concentration (75 mg of carvacrol/L). Moreover, a high-forage (F) and a high-concentrate (C) diet were considered because diet-dependent effects have been observed in previous studies^2^.

In agreement with the latter statement, NEO caused a slight but significant inhibition of ruminal fermentation in the vials with the C diet as the total VFA production was decreased (*P* < 0.05) when compared to the control (Table 2). However, similar to our previous trial^29^, NEO did not affect the total VFA concentration in the F diet. In agreement with Macheboeuf et al.^30^, the VFA profile was altered as the propionate molar proportion (a gluconeogenic precursor for ruminants) was decreased (*P* < 0.05) with NEO and carvacrol (Table 1), probably due to the effects on some specific bacterial genera^31,32^, as explained below. Moreover, both caproate proportion and the Ac: Pr ratio were increased only when NEO, SEO and carvacrol were added to the vials of the F diet (Table 1), suggesting that carvacrol is the main compound responsible for modifying the VFA proportions, as previously described by other authors^2^.

The essential oils and their compounds, especially carvacrol, had an impact on what has been defined as the rumen core microbiota population, causing a statistically significant decrease in *Blautia,* a genus of anaerobic bacteria belonging to the family *Lachnospiraceae* (Clostridia class), which is involved, as cellulolytic bacteria, in the breakdown of cellulose and the consequent release of VFA such as butyrate^33^. *Butyricicoccus* is another strictly anaerobic bacterium (family *Oscillospiraceae*, Clostridia class) that produces high levels of butyrate and was significantly reduced with these treatments. However, the proportions of butyrate and caproate were significantly increased in several EO treatments, whereas the total VFA levels were not significantly modified (except for the NEO treatment). These apparently contradictory results between the ruminal in vitro fermentation parameters and the microbiota might be explained by the modification of the metabolic activity of ruminal bacteria, regardless of the changes in the relative abundance of the OTUs previously mentioned^34^. Likewise, it is plausible that other unsequenced microorganisms, such as protozoa, also involved in butyrate production, were somehow affected by the treatment.

Other taxa involved in starch and glucose fermentation, such as *Marinilactobacillus* and *Atopobium*, both of which produce lactic acid, were reduced by either *p*-cymene or γ-terpinene, with no significant effects on ruminal fermentation parameters, probably due to mild changes in OTUs, unable to achieve a significant effect on the total VFA level. However, *Marinilactobacillus* is a genus in the phylum Bacteroidetes, involved in carbohydrate fermentation leading to propionate, and therefore, the decreased observed in these bacteria might be at least partially related to the decrease in propionate and the increase in the Ac: Pr ratio observed in the treatment (Table 1). In any case, the effects of EO treatments on rumen fermentation patterns were limited and of little relevance from the nutritional point of view.

As far as methane production is concerned, no effects were observed when 75 mg/L of carvacrol (NEO or SEO treatments) or the individual compounds were added to the vials containing the F or the C diet. The effects of NEO and the components evaluated are dose-dependent, and according to the concentrations evaluated in the current trial, these compounds would not be expected to have a significant effect on methane production^30,35^, especially considering the lack of inhibition of ruminal fermentation (e.g., no significant changes in total VFA and the proportion of acetate- or gas production, *P* > 0.05). Although the sequencing strategy was not specifically designed to target Archaea, we identified Euryarchaeota at the phylum level (2.75%, see Supplementary Fig. S1 online) and *Methanobrevibacter* at the genus level (2.81%, Fig. 1). However, statistical significance was not observed at the genus level when comparing differentially abundant taxa by treatment (Table 3), which agrees with the lack of effects of the different compounds tested on methane production (Tables 1, 2). Higher concentrations of EOs reduce the ruminal protozoa population (not accounted for in this experiment) and hence methanogenesis^36^.

Regarding protein metabolism by rumen bacteria, the increased ammonia N concentration (NH_3_-N) observed when NEO and SEO were added to the vials containing the F diet suggests that proteolysis and bacterial lysis might have been increased or microbial protein synthesis reduced with these treatments. It must be kept in mind that the relative abundances of two different γ-proteobacteria, *Pantoea* sp.^37^ and *Psychrobacter*^38^, were increased with carvacrol, but the increased NH_3_-N concentration with NEO and SEO could not be attributed to this compound. In agreement with Busquet et al.^39^, carvacrol had no effect on NH_3_-N when added to the vials containing the F diet (Table 1). Nevertheless, carvacrol affects in vitro ruminal fermentation in a dose-dependent manner, and higher concentrations than the one used in this study have caused a reduction of in vitro ruminal protein degradation^30,40^.

Conversely, the significant increase in NH_3_-N observed when NEO or SEO were added to the F diet could be promoted by a combination of compounds^36^. Accordingly, Ultee et al.^41^ described synergistic effects against some microorganisms when *p*-cymene was combined with carvacrol. These effects were explained by the accumulation of *p*-cymene in the bacterial cytoplasmic membrane, thus causing its expansion and allowing both leakage of ions and transport across the bacterial cytoplasmic membrane of other compounds with antimicrobial effects (e.g., carvacrol). Therefore, bacterial lysis might have provoked an increment of NH_3_-N in this short 24-h incubation period, regardless of the fact that the reduction of proteolytic bacteria could promote a decrease in proteolytic activity (and a subsequent decline in NH_3_-N levels) in the mid-term. However, it is remarkable that the number of sequences *per* sample was reduced for the SEO treatment and the pure bioactive compounds but not for NEO, and therefore, the significant increase in NH_3_-N observed for NEO and SEO was probably caused by different mechanisms, as explained before. In any case, the significant increase in NH_3_-N observed when NEO or SEO were added to the vials containing the F diet should be kept in mind because if this effect was reproduced in vivo (far beyond the scope of this study), NH_3_-N would be transformed into urea in the liver and eliminated in urine or milk, thus impairing the nitrogen use efficiency of the animals.

Other remarkable effects were observed regarding the ruminal microbiota. For example, the predominance of Firmicutes and Bacteroidetes, followed by Proteobacteria and, at long distance, other phyla, confirms previous reported data^42,43^ and adds a detailed vision on the rumen microbiota response to a 24-h incubation with the tested EOs.

The F:B ratio, which strongly correlates with lipid metabolism and, consequently, with milk fat yield in cows^16^, was not modified by the treatments in this study. Furthermore, the limited effects of EO treatments on ruminal fermentation were confirmed by the lack of effects on the beta-diversity analysis and most alpha-diversity indices. However, two of the five treatments (e.g., carvacrol and SEO) affected the within-sample (alpha) microbial diversity. Particularly, carvacrol induced significant changes in eight genera included in taxa known as core rumen microbiota, as reported previously by other authors^44–47^. Remarkably, one of these changes was the increase in the *Candidatus Saccharimonas* population, a potential cellulose user that causes laminitis when occurring at high abundances in the ruminal microbiota of dairy cows^48,49^. Other genera with significant changes in the present study, such as *Blautia*^32^ and *Atopobium*^50^, are predominant in diets inducing subacute ruminal acidosis (SARA). However, in the present study, the abundance of *Blautia* (but not *Atopobium*) was decreased when ruminal fluid was incubated with carvacrol. Given the correlation between SARA and laminitis, these results are intriguing and suggest that the effects of carvacrol on animal health should be further investigated in ruminants. Finally, *Campylobacter,* an important human foodborne pathogen that colonises the gastrointestinal tract of cattle, was significantly reduced with carvacrol.

According to the results observed in the present study, NEO (when used at a concentration of 75 mg carvacrol/L) may reduce the propionate molar proportion and increase the NH_3_-N concentration at the ruminal level, with no differences in methane production when using high-forage diets. Mild effects on ruminal microbiota were promoted, mainly by carvacrol, which affected some genera related to subacute ruminal acidosis and laminitis as well as foodborne pathogens. Therefore, even though minor nutritional or environmental benefits can be expected, further research would be necessary to clarify the effects of *T. capitata* EO on animal health and microbial food safety.

## Supplementary Information

Supplementary Legends.

Supplementary Figure S1.

Supplementary Table S1.

Supplementary Table S2.

Supplementary Table S3.

Supplementary Table S4.

## Data Availability

The 16S rRNA gene sequences obtained from this study were deposited in the EMBL-EBI European Nucleotide Archive (ENA) repository with the accession number PRJEB57398. The rest of data are available upon request.
