# Supplementary material for: Effects of Thymbra capitata essential oil on in vitro fermentation end-products and ruminal bacterial communities
Source: Sci Rep. 2023 Mar 13;13:4153. doi: 10.1038/s41598-023-31370-9 (PMC10011596; doi:10.1038/s41598-023-31370-9)
Supplement: Supplementary file 1 — Supplementary Legends. [file 41598_2023_31370_MOESM1_ESM.docx]

**Supplementary Figure S1**. Description of the *in vitro* core rumen microbiota. Piechart of the relative abundance of OTUs at phyla level in the rumen core microbiota (OTUs shared by 100% of the samples). Phyla with relative abundance <1% have been grouped into "Lower than 1%".
