# Supplementary figures and images for: Effects of Thymbra capitata essential oil on in vitro fermentation end-products and ruminal bacterial communities
Source: Sci Rep. 2023 Mar 13;13:4153. doi: 10.1038/s41598-023-31370-9 (PMC10011596; doi:10.1038/s41598-023-31370-9)

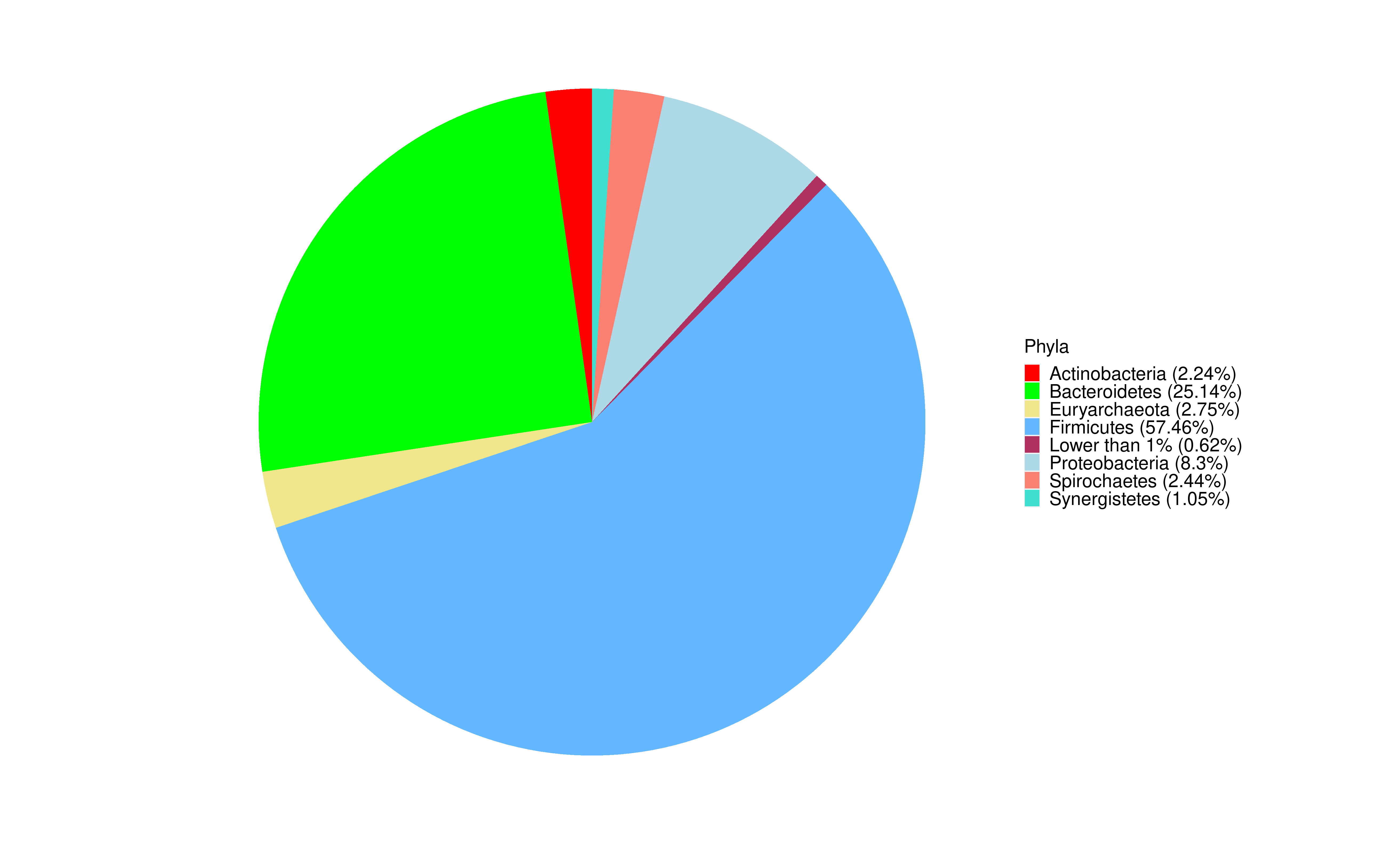

Supplement: Supplementary file 2 — Supplementary Figure S1. [file 41598_2023_31370_MOESM2_ESM.tif]
