# Supplementary material for: Effects of Thymbra capitata essential oil on in vitro fermentation end-products and ruminal bacterial communities
Source: Sci Rep. 2023 Mar 13;13:4153. doi: 10.1038/s41598-023-31370-9 (PMC10011596; doi:10.1038/s41598-023-31370-9)
Supplement: Supplementary file 3 — Supplementary Table S1. [file 41598_2023_31370_MOESM3_ESM.docx]

**Supplementary Table S1**. The core rumen microbiota consisted of 308 OTUs shared by 100% of the samples at the genus level.

| **Genus** | **Counts** |
| --- | --- |
| Uncultured or unknown | 0.14320615 |
| Prevotella 1 | 0.05515666 |
| Christensenellaceae R-7 group | 0.04505077 |
| Rikenellaceae RC9 gut group | 0.04316827 |
| Prevotella 9 | 0.02469724 |
| Streptococcus | 0.02243831 |
| Lachnospiraceae NK3A20 group | 0.0202112 |
| Escherichia-Shigella | 0.01961323 |
| Ruminococcaceae UCG-010 | 0.0195949 |
| Prevotella 7 | 0.01844588 |
| Treponema 2 | 0.01718553 |
| Butyrivibrio 2 | 0.01695455 |
| Ruminococcus 1 | 0.01685464 |
| Ruminococcaceae UCG-014 | 0.01614885 |
| Methanobrevibacter | 0.01487237 |
| Roseburia | 0.01437743 |
| Succiniclasticum | 0.01407248 |
| Ruminococcaceae NK4A214 group | 0.01396401 |
| Bacteroides | 0.01382642 |
| Succinivibrionaceae UCG-001 | 0.0126772 |
| Ruminococcus 2 | 0.01119179 |
| Prevotellaceae UCG-001 | 0.01110284 |
| Clostridium sensu stricto 1 | 0.01071952 |
| Olsenella | 0.01060154 |
| Shuttleworthia | 0.00927604 |
| Syntrophococcus | 0.00914251 |
| [Eubacterium] coprostanoligenes group | 0.00872368 |
| [Eubacterium] nodatum group | 0.00802857 |
| Oribacterium | 0.00788607 |
| Lachnospiraceae NK4A136 group | 0.00764053 |
| Ruminococcaceae UCG-005 | 0.00758992 |
| Pseudobutyrivibrio | 0.00746453 |
| Fibrobacter | 0.00734948 |
| [Eubacterium] eligens group | 0.0068571 |
| Lachnospira | 0.00675952 |
| Lysinibacillus | 0.00673153 |
| [Eubacterium] ruminantium group | 0.00622816 |
| Fusobacterium | 0.00612938 |
| Lachnospiraceae FCS020 group | 0.00597661 |
| Lachnospiraceae UCG-008 | 0.00594039 |
| Pyramidobacter | 0.00545624 |
| Ruminococcaceae UCG-002 | 0.00530784 |
| Desulfovibrio | 0.00530609 |
| [Ruminococcus] gauvreauii group | 0.00513134 |
| Acetitomaculum | 0.00508929 |
| Methanosphaera | 0.00501887 |
| Succinivibrio | 0.00496396 |
| Erysipelotrichaceae UCG-002 | 0.00461635 |
| Mitsuokella | 0.00458066 |
| Candidatus Saccharimonas | 0.00447461 |
| Family XIII AD3011 group | 0.00433165 |
| Sphaerochaeta | 0.00425039 |
| Prevotellaceae UCG-004 | 0.00420298 |
| Saccharofermentans | 0.00412261 |
| [Eubacterium] xylanophilum group | 0.00411411 |
| U29-B03 | 0.00398605 |
| Sharpea | 0.00384503 |
| Erysipelotrichaceae UCG-007 | 0.00374447 |
| Pseudomonas | 0.0037077 |
| Psychrobacter | 0.00339361 |
| Ruminococcaceae UCG-004 | 0.00319959 |
| CAG-352 | 0.00311798 |
| Anaerovorax | 0.0030956 |
| Ruminococcaceae UCG-009 | 0.00307456 |
| Dialister | 0.00287887 |
| Megasphaera | 0.00285171 |
| [Eubacterium] ventriosum group | 0.00285089 |
| Lachnospiraceae UCG-001 | 0.00278086 |
| FD2005 | 0.00271807 |
| Moryella | 0.00270742 |
| Agathobacter | 0.00260266 |
| Lachnoclostridium 1 | 0.00255349 |
| Campylobacter | 0.00251322 |
| [Bacteroides] pectinophilus group | 0.00250145 |
| Fretibacterium | 0.00244762 |
| Lachnospiraceae UCG-004 | 0.00242383 |
| Erysipelotrichaceae UCG-009 | 0.00239792 |
| Phascolarctobacterium | 0.00236704 |
| XBB1006 | 0.0023631 |
| Lachnoclostridium | 0.00232113 |
| Candidatus Methanomethylophilus | 0.00228499 |
| Bacillus | 0.0022593 |
| Prevotellaceae UCG-003 | 0.00219278 |
| Mailhella | 0.00211216 |
| [Eubacterium] oxidoreducens group | 0.00211118 |
| Papillibacter | 0.00207127 |
| Bifidobacterium | 0.00205238 |
| Lachnospiraceae AC2044 group | 0.0020276 |
| Lactobacillus | 0.00198328 |
| Atopobium | 0.00193404 |
| Acidaminococcus | 0.00190191 |
| Solibacillus | 0.00188666 |
| Ruminobacter | 0.00185183 |
| Ruminococcaceae UCG-013 | 0.00182738 |
| Erysipelotrichaceae UCG-004 | 0.00182078 |
| Parasutterella | 0.00181699 |
| Lachnospiraceae UCG-006 | 0.0017758 |
| Basfia | 0.00177139 |
| Erysipelotrichaceae UCG-006 | 0.00175547 |
| Catonella | 0.00174733 |
| Mogibacterium | 0.00171174 |
| Acinetobacter | 0.00170541 |
| Gallibacterium | 0.00169028 |
| Marvinbryantia | 0.00164898 |
| Pseudoramibacter | 0.00161619 |
| Coprococcus 2 | 0.00155358 |
| Alloprevotella | 0.00148453 |
| Lachnospiraceae NC2004 group | 0.00146407 |
| Anaerovibrio | 0.00145878 |
| [Acetivibrio] ethanolgignens group | 0.00139759 |
| [Eubacterium] cellulosolvens group | 0.00138891 |
| Ruminococcaceae UCG-003 | 0.00138669 |
| Howardella | 0.00138118 |
| Ruminiclostridium 5 | 0.00135732 |
| DNF00809 | 0.00134724 |
| Family XIII UCG-001 | 0.00130503 |
| Catenisphaera | 0.001299 |
| Butyricimonas | 0.00125084 |
| Lachnospiraceae XPB1014 group | 0.00121208 |
| Coprococcus 1 | 0.00119959 |
| Prevotellaceae YAB2003 group | 0.00118833 |
| rumen bacterium YS3 | 0.00118026 |
| Flexilinea | 0.00115107 |
| [Ruminococcus] torques group | 0.00114644 |
| Oscillospira | 0.00113092 |
| Turicibacter | 0.00108207 |
| Victivallis | 0.00104792 |
| [Eubacterium] hallii group | 0.00103472 |
| Alistipes | 0.00102722 |
| Solobacterium | 0.00100195 |
| Ruminococcaceae UCG-001 | 0.0009979 |
| GCA-900066225 | 0.00098572 |
| Globicatella | 0.0009608 |
| Anaerocolumna | 0.00095338 |
| Defluviitaleaceae UCG-011 | 0.0009398 |
| Anaerostipes | 0.00091681 |
| Vibrio | 0.00090075 |
| Lachnoclostridium 10 | 0.00089151 |
| Ruminiclostridium 6 | 0.00086831 |
| Cloacibacillus | 0.00085462 |
| UBA1819 | 0.00084183 |
| metagenome | 0.00082618 |
| Parabacteroides | 0.00082453 |
| Ruminococcaceae UCG-011 | 0.00081013 |
| Elusimicrobium | 0.00078472 |
| Z20 | 0.00076186 |
| Cellulosilyticum | 0.00075546 |
| Proteiniclasticum | 0.00075194 |
| Actinobacillus | 0.00074754 |
| Comamonas | 0.00074238 |
| Sutterella | 0.00073801 |
| Paenibacillus | 0.00073531 |
| Lactococcus | 0.00071118 |
| Asteroleplasma | 0.00069908 |
| Oscillibacter | 0.00069182 |
| Corynebacterium 1 | 0.00068349 |
| Anaerobacillus | 0.00067071 |
| Lachnospiraceae UCG-002 | 0.00066936 |
| Faecalibacterium | 0.00065123 |
| Desulfobulbus | 0.00064069 |
| Fermentimonas | 0.00063432 |
| Pseudoalteromonas | 0.00063142 |
| Lachnospiraceae UCG-009 | 0.00062775 |
| Caryophanon | 0.00061735 |
| Bacteroidales bacterium 55_9 | 0.00058795 |
| Glutamicibacter | 0.00057755 |
| Synergistes | 0.00054573 |
| Lachnospiraceae UCG-010 | 0.00052338 |
| Haemophilus | 0.00051314 |
| Kandleria | 0.00051313 |
| Enterococcus | 0.00051237 |
| Selenomonas 3 | 0.00050493 |
| Candidatus Soleaferrea | 0.00048824 |
| Ruminiclostridium 9 | 0.00046993 |
| Ruminococcaceae UCG-007 | 0.00043641 |
| Anaerosporobacter | 0.00043359 |
| Coprococcus 3 | 0.00042956 |
| Tyzzerella | 0.00042766 |
| Blautia | 0.00042378 |
| Clostridium sensu stricto 13 | 0.00040789 |
| Kurthia | 0.00040258 |
| Paeniglutamicibacter | 0.00040151 |
| [Eubacterium] brachy group | 0.00039367 |
| Lachnotalea | 0.0003927 |
| Herbinix | 0.00038802 |
| Coriobacteriaceae UCG-003 | 0.00038771 |
| Cetobacterium | 0.00037425 |
| p-1088-a5 gut group | 0.00036879 |
| Clostridium sensu stricto 11 | 0.00036417 |
| [Anaerorhabdus] furcosa group | 0.00035597 |
| Ruminiclostridium | 0.0003488 |
| Guggenheimella | 0.000346 |
| Subdoligranulum | 0.00034289 |
| Viridibacillus | 0.00034107 |
| Cutibacterium | 0.00034073 |
| Avibacterium | 0.00032808 |
| GCA-900066575 | 0.00032504 |
| Thiobacillus | 0.00031945 |
| Tannerella | 0.00031402 |
| Mesonia | 0.00031269 |
| Marinilactibacillus | 0.00030154 |
| Carnobacterium | 0.00029148 |
| Petrimonas | 0.00028613 |
| Robinsoniella | 0.00028135 |
| SP3-e08 | 0.00027781 |
| Sediminispirochaeta | 0.00027758 |
| Butyricicoccus | 0.00027109 |
| Propionibacterium | 0.00026287 |
| Aliivibrio | 0.00026257 |
| Epulopiscium | 0.00025087 |
| Alkaliphilus | 0.00024984 |
| Asaccharospora | 0.0002433 |
| Paraprevotella | 0.00023102 |
| Corynebacterium | 0.00022614 |
| Sediminibacillus | 0.00021747 |
| gut metagenome | 0.00020186 |
| Dorea | 0.00019528 |
| Catabacter | 0.00019178 |
| Lachnospiraceae FE2018 group | 0.00018999 |
| horsej-a03 | 0.00018998 |
| Clostridium sensu stricto 5 | 0.00018951 |
| Kocuria | 0.00018854 |
| Exiguobacterium | 0.00017594 |
| Enterorhabdus | 0.00017465 |
| Negativibacillus | 0.00016699 |
| Fastidiosipila | 0.00016635 |
| Proteiniphilum | 0.00016627 |
| Lachnospiraceae ND3007 group | 0.00016589 |
| Anaeroplasma | 0.00016071 |
| Ureibacillus | 0.00014967 |
| Brevibacterium | 0.00014489 |
| Lachnoclostridium 5 | 0.0001363 |
| Prevotellaceae NK3B31 group | 0.00013448 |
| Tectona grandis | 0.0001338 |
| probable genus 10 | 0.00013128 |
| Propioniciclava | 0.00013096 |
| Romboutsia | 0.00012932 |
| Solanum melongena (eggplant) | 0.00012579 |
| Raoultibacter | 0.00012215 |
| possible genus Sk018 | 0.00011936 |
| Clostridium sensu stricto 14 | 0.00011403 |
| Clostridium sensu stricto 4 | 0.00011206 |
| Tessaracoccus | 0.00011153 |
| Staphylococcus | 0.00010568 |
| Erysipelotrichaceae UCG-008 | 0.00010339 |
| Nicoletella | 0.00010181 |
| Okibacterium | 9.73E-05 |
| Winogradskyella | 9.64E-05 |
| Phycicoccus | 9.63E-05 |
| Selenomonas | 9.56E-05 |
| Clostridium sensu stricto 8 | 9.50E-05 |
| Angelakisella | 9.48E-05 |
| Anaerotruncus | 9.22E-05 |
| Ruminococcaceae V9D2013 group | 8.98E-05 |
| Allorhizobium-Neorhizobium-Pararhizobium-Rhizobium | 8.85E-05 |
| Enterobacter | 8.79E-05 |
| Ercella | 8.66E-05 |
| Rummeliibacillus | 8.52E-05 |
| Aerosphaera | 8.39E-05 |
| Veillonella | 8.34E-05 |
| Erysipelothrix | 7.80E-05 |
| Clostridioides | 7.79E-05 |
| Ruminococcaceae UCG-008 | 7.36E-05 |
| Ornithinicoccus | 7.34E-05 |
| Alkalibacterium | 7.23E-05 |
| Sporosarcina | 7.18E-05 |
| Klebsiella | 7.15E-05 |
| Ruminiclostridium 1 | 7.01E-05 |
| Terrisporobacter | 6.99E-05 |
| Intestinimonas | 6.91E-05 |
| [Eubacterium] saphenum group | 6.63E-05 |
| Eubacterium | 6.55E-05 |
| Paraburkholderia tropica | 6.48E-05 |
| Frederiksenia | 6.09E-05 |
| Fontibacillus | 6.03E-05 |
| Pantoea | 5.85E-05 |
| [Ruminococcus] gnavus group | 5.62E-05 |
| Halomonas | 5.57E-05 |
| Clostridium sensu stricto 3 | 5.55E-05 |
| Planomicrobium | 5.48E-05 |
| Schwartzia | 5.46E-05 |
| Denitrobacterium | 5.40E-05 |
| Truepera | 4.97E-05 |
| Bibersteinia | 4.80E-05 |
| Geosporobacter | 4.79E-05 |
| 28-apr | 4.73E-05 |
| Prevotella 6 | 4.71E-05 |
| Sphingomonas | 4.59E-05 |
| W5053 | 4.25E-05 |
| Peptostreptococcus | 4.05E-05 |
| Leucobacter | 4.04E-05 |
| Methanomicrobium | 3.98E-05 |
| Sarcina | 3.95E-05 |
| Methylomicrobium | 3.87E-05 |
| Helcococcus | 3.73E-05 |
| Citrobacter | 3.63E-05 |
| Lactonifactor | 3.62E-05 |
| Bhargavaea | 3.60E-05 |
| Anaerofustis | 3.58E-05 |
| Escherichia coli | 3.53E-05 |
| Tyzzerella 3 | 3.52E-05 |
| Prevotella | 3.49E-05 |
| Pseudoscardovia | 3.40E-05 |
| Kosakonia | 3.31E-05 |
| Vagococcus | 3.18E-05 |
| Blastopirellula | 2.84E-05 |
| Moraxella | 1.65E-05 |
| Pasteurella | 1.02E-05 |
