# Supplementary material for: Effects of Thymbra capitata essential oil on in vitro fermentation end-products and ruminal bacterial communities
Source: Sci Rep. 2023 Mar 13;13:4153. doi: 10.1038/s41598-023-31370-9 (PMC10011596; doi:10.1038/s41598-023-31370-9)
Supplement: Supplementary file 4 — Supplementary Table S2. [file 41598_2023_31370_MOESM4_ESM.docx]

**Supplementary Table S2.** Average values of alpha diversity indices per treatment

| **treatment** | **N** | **chao1** | **ace** | **fisher_alpha** | **observed_otus** | **Shannon** | **Simpson** | **equitability** | **simpson_e** |
| --- | --- | --- | --- | --- | --- | --- | --- | --- | --- |
| Control | 8 | 1527.47 | 1501.14 | 644.98 | 1136.88 | 9.79 | 0.999 | 0.965 | 0.642 |
| NEO | 8 | 1535.38 | 1505.57 | 643.91 | 1133.00 | 9.79 | 0.999 | 0.965 | 0.639 |
| SEO | 8 | 1178.94 | 1183.46 | 523.44 | 904.25 | 9.42 | 0.998 | 0.968 | 0.666 |
| carvacrol | 8 | 1236.80 | 1229.39 | 536.83 | 934.63 | 9.39 | 0.998 | 0.967 | 0.668 |
| *p*-cymene | 8 | 1402.46 | 1351.16 | 582.55 | 995.75 | 9.60 | 0.998 | 0.965 | 0.645 |
| γ-terpinene | 8 | 1372.80 | 1335.32 | 580.38 | 1015.38 | 9.64 | 0.998 | 0.966 | 0.650 |
