# Supplementary material for: Effects of Thymbra capitata essential oil on in vitro fermentation end-products and ruminal bacterial communities
Source: Sci Rep. 2023 Mar 13;13:4153. doi: 10.1038/s41598-023-31370-9 (PMC10011596; doi:10.1038/s41598-023-31370-9)
Supplement: Supplementary file 5 — Supplementary Table S3. [file 41598_2023_31370_MOESM5_ESM.docx]

**Supplementary Table S3.** Differentially abundant taxa by treatment. The table presents the counts and p-values for every taxon with a significant differential abundance after different compounds' co-incubation. P-values are presented for each treatment against Control, and overall P-value represents the significance of the influence of the treatment against the null hypothesis that no counts would have not to be influenced by treatment.

| **level** | **Taxa** | **Control** | **carvacrol** |  | **γ-terpinene** |  | **NEO** |  | ***p*-cymene** |  | **SEO** |  | **Overall P-value** |
| --- | --- | --- | --- | --- | --- | --- | --- | --- | --- | --- | --- | --- | --- |
|  |  | Relative abundance (%) | Relative abundance (%) | P-value | Relative abundance (%) | P-value | Relative abundance (%) | P-value | Relative abundance (%) | P-value | Relative abundance (%) | P-value |  |
| phylum | Firmicutes | 54.365 | 58.147 | **0.010** | 52.632 | 0.059 | 55.299 | 0.834 | 54.976 | 0.061 | 169800.318 | **0.010** | **0.012** |
| phylum | Patescibacteria | 0.368 | 0.796 | **0.035** | 0.385 | 0.777 | 0.462 | 0.265 | 0.310 | 0.199 | 1309.451 | 0.693 | **0.019** |
| phylum | Synergistetes | 1.024 | 0.890 | **0.012** | 0.999 | 0.194 | 1.022 | 0.922 | 0.949 | **0.044** | 2877.788 | **0.013** | **0.027** |
| phylum | Actinobacteria | 1.994 | 2.575 | 0.193 | 1.843 | 0.074 | 2.189 | 0.285 | 1.948 | 0.060 | 6909.890 | 0.242 | **0.030** |
| phylum | Epsilonbacteraeota | 0.278 | 0.167 | **0.008** | 0.295 | 0.759 | 0.247 | 0.484 | 0.305 | 0.601 | 686.830 | **0.024** | **0.036** |
| class | Saccharimonadia | 0.344 | 0.771 | **0.030** | 0.377 | 0.953 | 0.435 | 0.255 | 0.284 | 0.211 | 1267.752 | 0.829 | **0.021** |
| class | Coriobacteriia | 1.580 | 1.589 | 0.059 | 1.405 | 0.092 | 1.778 | 0.283 | 1.513 | 0.094 | 5269.435 | 0.220 | **0.024** |
| class | Synergistia | 1.024 | 0.890 | **0.012** | 0.999 | 0.194 | 1.022 | 0.922 | 0.949 | **0.044** | 2877.788 | **0.013** | **0.027** |
| class | Campylobacteria | 0.278 | 0.167 | **0.008** | 0.295 | 0.759 | 0.247 | 0.484 | 0.305 | 0.601 | 686.830 | **0.024** | **0.036** |
| class | Clostridia | 43.060 | 47.280 | **0.032** | 42.110 | 0.108 | 44.888 | 0.605 | 43.985 | 0.100 | 139953.598 | **0.045** | **0.037** |
| order | Saccharimonadales | 0.344 | 0.771 | **0.030** | 0.377 | 0.953 | 0.435 | 0.255 | 0.284 | 0.211 | 1267.752 | 0.829 | **0.021** |
| order | Coriobacteriales | 1.580 | 1.589 | 0.060 | 1.405 | 0.092 | 1.778 | 0.282 | 1.513 | 0.094 | 5269.435 | 0.220 | **0.024** |
| order | Bacillales | 2.425 | 1.291 | **0.015** | 1.578 | 0.090 | 0.742 | **0.008** | 2.070 | 0.304 | 1917.815 | **0.003** | **0.027** |
| order | Synergistales | 1.024 | 0.980 | **0.012** | 0.999 | 0.194 | 1.022 | 0.922 | 0.949 | **0.044** | 2877.788 | **0.013** | **0.027** |
| order | Campylobacterales | 0.278 | 0.167 | **0.008** | 0.295 | 0.759 | 0.247 | 0.484 | 0.305 | 0.601 | 686.830 | **0.024** | **0.036** |
| order | Clostridiales | 43.060 | 47.280 | **0.032** | 42.110 | 0.108 | 44.888 | 0.505 | 43.985 | 0.100 | 139953.598 | **0.045** | **0.037** |
| order | Pirellulales | 0.071 | 0.036 | 0.097 | 0.073 | 0.874 | 0.023 | **0.038** | 0.025 | **0.030** | 36.787 | **0.009** | **0.038** |
| family | Family XIII | 1.860 | 2.168 | **0.028** | 1.789 | 0.114 | 1.924 | 0.711 | 1.734 | **0.017** | 5694.219 | **0.019** | **0.014** |
| family | Planococcaceae | 1.871 | 0.556 | **0.007** | 1.217 | 0.098 | 0.552 | **0.009** | 1.659 | 0.356 | 1100.453 | **0.002** | **0.016** |
| family | Saccharimonadaceae | 0.338 | 0.765 | **0.029** | 0.373 | 0.965 | 0.427 | 0.268 | 0.279 | 0.221 | 1257.559 | 0.861 | **0.023** |
| family | Synergistaceae | 1.024 | 0.890 | **0.012** | 0.999 | 0.194 | 1.022 | 0.922 | 0.949 | **0.044** | 2877.788 | **0.013** | **0.027** |
| family | Victivallaceae | 0.359 | 0.318 | 0.059 | 0.344 | 0.272 | 0.336 | 0.674 | 0.301 | **0.020** | 864.263 | **0.005** | **0.034** |
| family | Eggerthellaceae | 0.161 | 0.170 | 0.488 | 0.090 | 0.070 | 0.227 | 0.133 | 0.160 | 0.492 | 446.371 | 0.264 | **0.034** |
| family | Campylobacteraceae | 0.278 | 0.167 | **0.008** | 0.295 | 0.759 | 0.247 | 0.484 | 0.305 | 0.601 | 686.830 | **0.024** | **0.036** |
| family | Pirellulaceae | 0.071 | 0.036 | 0.097 | 0.073 | 0.874 | 0.023 | **0.038** | 0.025 | **0.030** | 36.787 | **0.009** | **0.038** |
| family | Carnobacteriaceae | 0.071 | 0.087 | 0.228 | 0.073 | **0.011** | 0.038 | 0.059 | 0.091 | 0.763 | 195.726 | 0.249 | **0.043** |
