# Supplementary material for: Effects of Thymbra capitata essential oil on in vitro fermentation end-products and ruminal bacterial communities
Source: Sci Rep. 2023 Mar 13;13:4153. doi: 10.1038/s41598-023-31370-9 (PMC10011596; doi:10.1038/s41598-023-31370-9)
Supplement: Supplementary file 6 — Supplementary Table S4. [file 41598_2023_31370_MOESM6_ESM.docx]

**Supplementary Table S4**. F:B ratio by treatment: original values, estimated differences (benchmark is Control), P-values, and bootstrapped averages.

RSE = relative standard error

| **treatment** | **B_avg** | **F_avg** | **FB_avg** | **FB_med** | **estimate_diff** | **RSE** | **P-value** | **boot_avg_FB** | **boot_med_FB** |
| --- | --- | --- | --- | --- | --- | --- | --- | --- | --- |
| Control | 904.016 | 2102.751 | 2.361 | 2.339 |  |  |  | 2.342 | 2.339 |
| NEO | 889.186 | 2133.608 | 2.493 | 2.356 | 0.132 | 0.957 | 0.891 | 2.381 | 2.356 |
| SEO | 656.792 | 1699.391 | 3.536 | 2.385 | 1.175 | 0.957 | 0.226 | 2.549 | 2.385 |
| carvacrol | 734.557 | 1695.336 | 3.867 | 2.045 | 1.506 | 0.957 | 0.123 | 2.541 | 2.045 |
| *p*-cymene | 765.902 | 1813.158 | 2.396 | 2.519 | 0.035 | 0.957 | 0.971 | 2.446 | 2.519 |
| γ-terpinene | 861.617 | 1811.559 | 2.144 | 2.077 | -0.217 | 0.957 | 0.821 | 2.085 | 2.077 |

RSE = relative standard error
